# Variations in vitamin D status among Chinese children aged 1–6 years during the COVID-19 pandemic

**DOI:** 10.3389/fpubh.2025.1514355

**Published:** 2025-01-23

**Authors:** Yongfeng Qiao, Xiaoqin Wang, Yanfen Ma, Jian Hu

**Affiliations:** ^1^Department of Clinical Laboratory, Hanzhong Central Hospital, Hanzhong, China; ^2^Department of Clinical Laboratory, The First affiliated hospital of Xi’an Jiaotong University, Xi'an, China

**Keywords:** 25-hydroxyvitamin D, 25-hydroxyvitamin D_2_, 25-hydroxyvitamin D_3_, LC–MS/MS, children, COVID-19

## Abstract

**Background:**

Vitamin D deficiency has been a critical global health issue within the pediatric population. Closed-off management brought about by the COVID-19 pandemic has drastically impacted outdoor activities and sunlight exposure, however, whether it indirectly further exacerbated the vitamin D deficiency has not been largely investigated, especially among children in China. The purpose of this study was to evaluate 25(OH)D concentrations in children before and during the COVID-19 lockdown and to analyze the factors influencing their vitamin D status.

**Methods:**

A cross-sectional survey included children aged 1–6 years from Han Zhong Central Hospital in the southern Shanxi Province of China. This study examined healthy children from a pediatric health care department over two periods: before COVID-19 (March 2019–February 2020), and during COVID-19 (March 2020–February 2021). Total 25(OH)D concentrations were compared between the two observation periods. Vitamin D status was determined by 25(OH)D concentrations: deficient (<20 ng/ml), insufficient (20–29 ng/ml), and sufficient (30–100 ng/ml).

**Results:**

The study involved 6,780 children, with 52.8% being 1-year-olds, 23.1% being 2-year-olds, and 24.1% being 3 to 6-year-olds. Boys and girls were 52.8 and 47.2%, respectively. The actual prevalence of deficiency in vitamin D nutritional status among children was 2.8%, with 87.1% of cases in those aged 3 to 6 years. Vitamin D insufficiency was 18.3%, affecting 54.8% of the same demographic. The average of 25(OH)D concentration were 38.2 ± 9.8 ng/ml, significantly varying by age and season. 25(OH)D concentrations decreased with age, from 42.3 ± 8.8 ng/ml at 1-year-olds to 37.4 ± 8.2 ng/ml at 2-year-olds, and further to 30.2 ± 8.1 ng/ml at 3 to 6-year-olds. Seasonal variations showed that 25(OH)D concentrations were higher in spring (38.7 ± 10.1 ng/ml), summer (38.7 ± 10.0 ng/ml), and fall (38.6 ± 9.2 ng/ml) in comparison to winter (36.0 ± 9.8 ng/ml). Additionally, the concentrations of 25(OH)D in spring exhibited a decrease during the COVID-19 pandemic (37.9 ± 10.3 ng/ml) in comparison to the pre-pandemic measurements (39.3 ± 9.9 ng/ml) (*p* = 0.008), while winter concentrations increased from (35.1 ± 10.4 ng/ml) to (37.9 ± 10.3 ng/ml) during the pandemic (*p* = 0.002).

**Conclusion:**

The research indicated that vitamin D deficiency is uncommon among Chinese children, with 25(OH)D concentrations experiencing a notable decline in those aged 3–6 years. The findings suggested a potential need for tailored supplementation strategies and possibly higher doses for this age group, along with monitoring 25(OH)D concentrations to evaluate supplementation effectiveness. COVID-19-related restrictions minimally affected children’s 25(OH)D concentrations, revealing the nutritional implications of the pandemic.

## 1 Introduction

Vitamin D is associated with child growth and development, which exists in two forms: vitamin D2 and vitamin D3. Vitamin D2 is derived from ergosterol in yeasts, fungi, and sunlight-exposed mushrooms and is commonly added to supplements and fortified foods like milk and cereals (1). Vitamin D3 is primarily synthesized through sunlight exposure and can be obtained from vitamin D supplements, fish liver oil, egg yolks, dairy products, and fortified foods (2). A deficiency of vitamin D can lead to serious health issues, such as skeletal deformities from poor calcium absorption (3), weakened immunity (4, 5), increased infection risk (6), stunted growth, and potential mental health problems like depression and anxiety (7, 8). The lack of vitamin D in the pediatric population is a significant health issue globally (9), and China faces similar challenges. Research conducted across various regions of China reveals significant variations in the deficiency rates of vitamin D among pediatric populations. In Harbin, 55% of children aged 0 to 12 years old demonstrated various levels of deficiency or insufficiency when it comes to vitamin D (10), while in Wuxi, 48.1% of preschool children aged 0–6 lack vitamin D (11). In contrast, the proportion of deficiency and inadequacy of vitamin D among the pediatric population aged 0–4 is only 1.06% in Yunnan Province (12).

COVID-19 was originally distributed on a small scale and then disseminated rapidly around the world at an astonishing speed, leading to a pandemic being declared on March 11, 2020 (13). During the pandemic, various countries enforced strict closed-off management, resulting in unprecedented disruptions to children’s routines, including prolonged school closures and limited outdoor activities (5). As a result, children were largely housebound and had minimal exposure to sunlight. Consequently, these pandemic-related confinement measures potentially compromised children’s vitamin D synthesis, potentially impacting their 25(OH)D levels. There is still a considerable and important subject of ongoing debate among researchers regarding whether the COVID-19 pandemic restrictions affect people’s vitamin D status (14–17). However, limited information exists regarding the long-term health effects of large-scale disease outbreaks on children’s vitamin status in China following such interventions. Moreover, the reported results varied across different regions (18, 19).

Therefore, this study aimed to conduct a comprehensive analysis of the 25(OH)D concentrations and identify the various factors influencing vitamin D status among children aged 1 to 6 years in Hanzhong City, southern Shaanxi Province, China, to reveal the nutritional status in this region and provide a reference for future research on vitamin D nutrition. Simultaneously，it investigated the changes in 25(OH)D concentrations among children across groups stratified by age, sex, and season before and during the COVID-19 lockdown, and evaluated the effect on vitamin D status by the lockdown.

## 2 Methods

This study was a large-scale investigation conducted over two time periods: before COVID-19 (March2019-February 2020), and during COVID-19(March 2020–February 2021). The study involved 6,780 children aged 1 to 6 years from Hanzhong Central Hospital in Hanzhong, located in the southern Shanxi Province of China, with 3,666 cases from before the pre-COVID-19 period and 3,114 different cases during the pandemic. The study subjects were healthy children from the pediatric health department based on specific inclusion and exclusion criteria. Participants took a minimum of 400 IU of oral vitamin D supplements every day as advised by their doctors while monitoring their 25(OH)D concentrations, excluding those with recent gastrointestinal infections, liver, kidney, endocrine, metabolic disorders, and congenital defects.

The data were collected on the date of hospital visits, age, gender, serum 25(OH)D concentrations, 25(OH)D_3_ concentrations, and 25(OH)D_2_ concentrations from the Xin- he Laboratory Information System and exported to an Excel sheet. Blood samples from all children were withdrawn from the antecubital vein in the morning while fasting. Serum 25(OH)D_,_25(OH)D_3,_ and 25(OH)D_2_ concentrations were quantified on the AB SCIEX 3200MD Mass Spectrometry systems (Applied Biosystems, USA) through the application of liquid chromatography–tandem mass spectrometry. Serum 25(OH)D concentration is the combined 25(OH)D_3_ and 25(OH)D_2_ values. The research protocol received ethical approval from the Ethics Committee of Hanzhong Central Hospital (IRB2019-Y), and was conducted in strict adherence to the ethical principles of the Declaration of Helsinki (2013 revision). Given the retrospective design of this investigation, the Institutional Review Board granted a waiver to obtain individual informed consent.

According to the latest clinical practice guidelines on vitamin D published online in 2024 by the research team of Michael et al. (20), the status of vitamin D in individuals was determined by 25(OH)D concentrations: deficient (<20 ng/ml), insufficient (20–29 ng/ml), and sufficient (30–100 ng/ml). Age was categorized into three groups: 1-year-olds, 2-year-olds, and 3 to 6-year-olds. Gender was categorized as either boy or girl. The following standards organized the seasons: spring includes March, April, and May; summer consists of June, July, and August; fall covers September, October, and November; and winter comprises December, January, and February, according to the dates of sample collection.

### 2.1 Statistical analyses

All Statistics analyses for this study were performed utilizing IBM SPSS Statistical software version 22, a software product developed by IBM Corp. of Armonk, New York. The graphs were plotted using OriginLab software version OriginPro 2023 (OriginLab Corporation, Northampton, MA, USA). The concentrations of 25(OH)D, 25(OH)D_3,_ and 25(OH)D_2_ were presented as means and standard deviations, compared between season and age groups using one-way analysis of Variance and gender groups using an independent t-test. Additionally, an independent t-test was performed for comparison of 25(OH)D concentrations in all children before and during the COVID-19. Categorical variables were presented in the form of relative frequencies, and comparisons were performed using the χ^2^ tests. A two-tailed *p*-value <0.05 was considered statistically significant.

## 3 Results

Table 1 summarized the general demographic characteristics of all children. The study included 6,780 children, with 52.8% aged 1, 23.1% aged 2, and 24.1% aged 3–6. The gender distribution was nearly balanced, with 52.8% boys and 47.2% girls. Seasonally, summer recruitment was highest at 34.8%, compared to spring (24.0%), fall (25.8%), and winter (15.4%). Additionally, 54.1% of participants were enrolled before the COVID-19 pandemic, while the remaining 45.9% recruited during the pandemic.

**Table 1:** Baseline characteristics of children (*n* = 6,780).

	*N* (%)	*N* (%)	
		Pre-COVID-19	COVID-19
All Children	6,780(100%)	3,666 (54.1%)	3,114(45.9%)
Age			
1 years-old	3,580(52.8%)	1,837 (50.1%)	1,743(61.5%)
2 years-old	1,565(23.1%)	923 (25.2%)	642(23.6%)
3–6 years-old	1,635(24.1%)	906 (24.7%)	729 (14.8%)
Gender			
Boy	3,583(52.8%)	1938 (48.4%)	1,645 (53.3%)
Girl	3,197(47.2%)	1,728 (51.6%)	1,469 (46.7%)
Season			
Spring	1,628(24.0%)	972 (23.1%)	656 (24.3%)
Summer	2,360(34.8%)	1,227 (24.2%)	1,133 (34.8%)
Fall	1,746(25.8%)	941 (15.6%)	805 (26.6%)
Winter	1,046(15.4%)	526 (37.1%)	520 (14.2%)

Table 2 provided a detailed report on the prevalence rates of vitamin D, revealing that the rates were 2.8% for deficiency and 18.3% for insufficiency, respectively. Among those deficient, 87.1% were aged 3 to 6-years-olds, significantly higher than 0.5% in 1-year-olds and 12.4% in 2-year-olds (*p* < 0.001). Winter showed a deficiency rate of 37.1%, markedly higher than other seasons (spring 23.1%, summer 24.2%, fall 15.6%) (*p* < 0.001). However, no statistical gender differences were determined. Similarly, vitamin D insufficiency was most prevalent in 3 to 6-year-olds (54.8%), differing significantly from 1-year-olds (22.9%) and 2-year-olds (22.3%) (*p* < 0.001). Additionally, insufficiency was higher in summer at 36.3% than in spring (22.8%), fall (23.5%), and winter (17.4%) (p < 0.001).

**Table 2:** The distribution of vitamin D in children.

	25(OH) D concentrations categories			*p*-value
	The prevalence of deficiency [25(OH)D < 20 ng/ml]	The prevalence of insufficiency [20 ≤ 25(OH)D ≤ 29 ng/ml]	The prevalence of sufficiency [30 ≤ 25(OH)D ≤ 100 ng/ml]	
All Children	186 (2.8%)	1,240(18.3%)	5,354(78.9%)	**<0.001** ^*^
Age				**<0.001** ^*^
1 year old	1 (0.5%)	284 (22.9%)	3,295 (61.5%)	
2 years old	23 (12.4%)	277 (22.3%)	1,265 (23.6%)	
3-6 years old	162 (87.1%)	679 (54.8%)	794 (14.8%)	
Gender				**0.265** ^*^
Boys	90 (48.4%)	640 (51.6%)	2,853 (53.3%)	
Girls	96 (51.6%)	600 (48.4%)	2,501 (46.7%)	
Season				**<0.001** ^*^
Spring	43 (23.1%)	283 (22.8%)	1,302 (24.3%)	
Summer	45 (24.2%)	450 (36.3%)	1865 (34.8%)	
Fall	29 (15.6%)	291 (23.5%)	1,426 (26.6%)	
Winter	69 (37.1%)	216 (17.4%)	761 (14.2%)	

* Chi-square tests were performed to compare 25(OH)D, 25(OH)D_2_, 25(OH)D_3_ distribution status stratified by age, gender and season.

**Figure 1:**
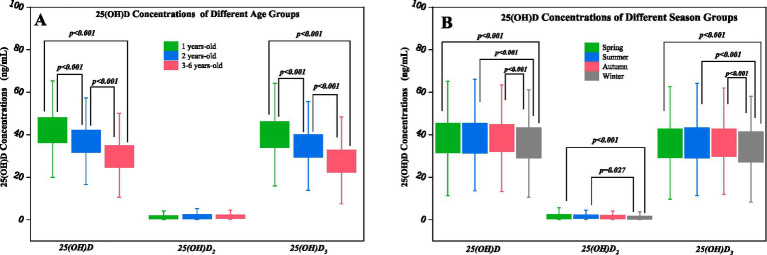
Comparison of 25(OH)D concentrations by age **(A)** and season **(B)**.

The average concentrations of 25(OH)D were (38.2 ± 9.8) ng/ml, 25(OH)D_2_ were (2.3 ± 3.9) ng/ml, and 25(OH)D_3_ were (35.9 ± 9.9) ng/ml. Among the concentrations of 25(OH)D among all children, statistical differences were detected between the age groups, the gender groups, and the season groups (*p* < 0.001, *p* = 0.009, p < 0.001). Similarly, the concentrations of 25(OH)D_3_ showed marked differences among the age groups and the season groups, respectively (*p* < 0.001), but no difference in the gender group (*p* > 0.05). Additionally, the comparison of 25(OH)D_2_ concentrations revealed a statistical difference by the season (*p* = 0.003), but no differences in the age groups or the gender groups (*p* > 0.05) (Table 3). The concentrations of 25(OH)D and 25(OH)D_3_ among all children were highest in the 1-year-old group (42.3 ± 8.8 ng/ml, 40.1 ± 9.0 ng/ml), followed by the 2-years-old group (37.4 ± 8.2 ng/ml, 34.9 ± 8.1 ng/ml), and were least in the 3 to 6-years-old group (30.2 ± 8.1 ng/ml,27.8 ± 8.1 ng/ml) (Table 3; Figure 1A). The concentrations of 25(OH)D of all participating children in the research collected in spring (38.7 ± 10.1 ng/ml), summer (38.7 ± 10.0 ng/ml), and fall (38.6 ± 9.2 ng/ml) were all higher than in winter (36.0 ± 9.8 ng/ml). Again, 25(OH)D_3_ concentrations were significantly higher during spring (36.2 ± 10.2 ng/ml), summer (36.3 ± 10.0 ng/ml), and fall (36.3 ± 9.4 ng/ml) in comparison to winter (34.0 ± 10.0 ng/ml). 25(OH)D_2_ concentrations collected in spring and summer (2.5 ± 4.4 ng/ml, 2.4 ± 3.8 ng/ml) were higher than in winter (2.0 ± 3.5 ng/ml) (Table 3; Figure 1B).

**Table 3:** Comparison of 25(OH)D concentrations among groups.

	25(OH)D (Mean ± SD)	*p*-value	25(OH)D_2_ (Mean ± SD)	*p*-value	25(OH)D_3_ (Mean ± SD)	*p*-value
All Children	38.2 ± 9.8		2.3 ± 3.9		35.9 ± 9.9	
Vitamin D status		**<0.001** ^*^		**<0.001** ^*^		**<0.001** ^*^
Deficiency	17.4 ± 2.3		1.2 ± 1.7		16.1 ± 2.6	
Insufficieny	26.3 ± 2.7		1.8 ± 2.5		24.5 ± 3.6	
Sufficiency	41.7 ± 7.8		2.5 ± 4.2		39.3 ± 8.2	
Age		**<0.001** ^*^		**0.054** ^*^		**<0.001** ^*^
1 year old	42.3 ± 8.8		2.2 ± 3.9		40.1 ± 9.0	
2 years old	37.4 ± 8.2		2.5 ± 4.3		34.9 ± 8.1	
3–6 years old	30.2 ± 8.1		2.3 ± 3.6		27.8 ± 8.1	
Gender		**0.009** ^**#**^		**0.085** ^**^#^**^		**0.575** ^**^#^**^
Boys	38.3 ± 9.6		2.4 ± 4.0		36.0 ± 9.9	
Girls	38.2 ± 10.1		2.3 ± 3.8		36.0 ± 10.0	
Season		**<0.001** ^*^		**0.003** ^*^		**<0.001** ^*^
Spring	38.7 ± 10.1		2.5 ± 4.4		36.2 ± 10.2	
Summer	38.7 ± 10.0		2.4 ± 3.8		36.3 ± 10.0	
Fall	38.6 ± 9.2		2.2 ± 3.9		36.3 ± 9.4	
Winter	36.0 ± 9.8		2.0 ± 3.5		34.0 ± 10.0	

* The ANOVA test was performed to compare 25(OH)D, 25(OH)D_2_, 25(OH)D_3_ concentrations stratified by Vitamin D status, age and season.

# The independent *t*-test was performed to compare 25(OH)D, 25(OH)D_2_, 25(OH)D_3_ concentrations stratified by gender.

**Figure 2:**
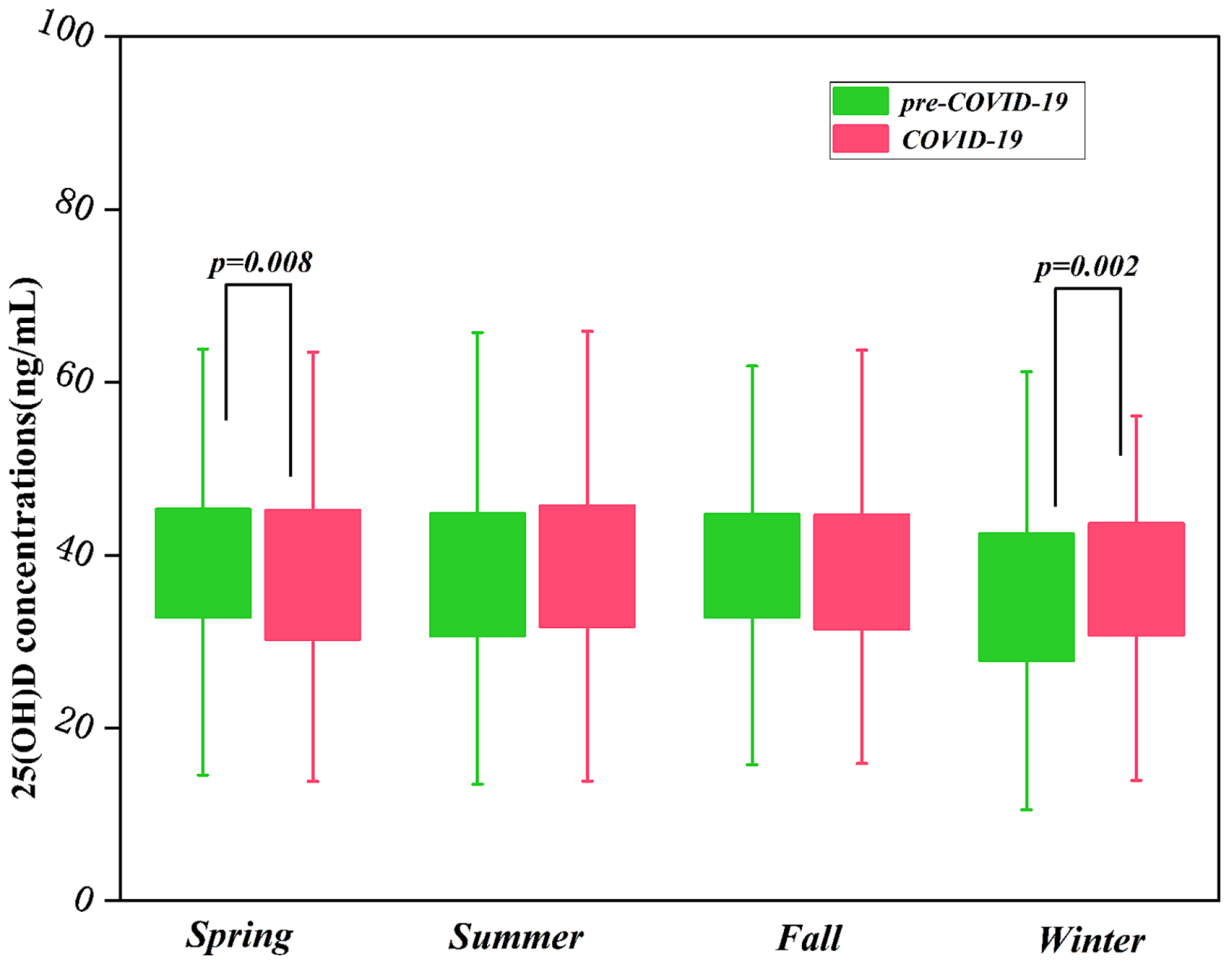
Seasonal comparison of 25(OH)D concentrations over two periods.

Table 4 presented a detailed comparison of 25(OH)D concentrations in children before and during the COVID-19 pandemic. When comparing the two different time periods, there was no statistical difference in the average concentrations of 25(OH)D (*p* > 0.05). Likewise, the comparison of average concentrations of 25(OH)D in all children divided by age and gender between the two periods were not substantially altered (p > 0.05) (Table 4). However, 25(OH)D concentrations in all children collected in spring during the pre-COVID-19 (39.3 ± 9.9 ng/ml) were higher than those in spring during the COVID-19 pandemic (37.9 ± 10.3 ng/ml) (*p* = 0.008); 25(OH)D concentrations in all individuals collected in winter during the pre-COVID-19 (35.1 ± 10.4 ng/ml) were lower than those in winter during COVID-19 pandemic (36.9 ± 9.0 ng/ml) (*p* = 0.002) (Table 4; Figure 2).

**Table 4:** Comparison of 25(OH)D concentrations over two periods.

	25(OH)D concentrations (Mean ± SD)		*p*-value^*^
	pre-COVID-19	COVID-19	
All Children	38.3 ± 10.0	38.2 ± 9.6	**0.791**
Age			
1 years-old	42.4 ± 9.1	42.2 ± 8.5	**0.548**
2 years-old	37.7 ± 8.4	37.0 ± 8.0	**0.113**
3–6 years-old	30.5 ± 8.5	29.7 ± 7.6	**0.035**
Gender			
Boys	38.5 ± 9.8	38.1 ± 9.3	**0.184**
Girls	38.0 ± 10.3	38.3 ± 10.0	**0.337**
Season			
Spring	39.3 ± 9.9	37.9 ± 10.3	**0.008**
Summer	38.4 ± 10.2	39.0 ± 9.8	**0.168**
Fall	38.9 ± 9.3	38.2 ± 9.1	**0.134**
Winter	35.1 ± 10.4	36.9 ± 9.0	**0.002**

* The independent *t*-test was performed to compare 25(OH)D, 25-hydroxyvitamin D concentrations.

## 4 Discussion

In this study, we used LC–MS/MS technology to investigate children’s 25(OH)D concentrations and influencing factors before and during COVID-19, investigating the effects of the pandemic on vitamin D status. We found that vitamin D deficiency is less severe in Chinese children. The concentrations of 25(OH)D gradually decrease with age. Additionally, we reported that the COVID-19-related restrictions minimally affected children’s 25(OH)D concentrations.

The study indicated that the actual incidence of deficiency in vitamin D nutritional status is extremely low, less than 3%, consistent with the studies reported by Wu et al. (12) in Yunnan Province and Yuan et al. (21) in Beijing regarding deficiency among pediatric population in China, which is lower than Schleicher (22) reported the deficiency rate among children aged 1–11 in the 2007–2010 NHANES. This significant finding is closely linked to China’s current public health policy on vitamin D supplementation, which forming a systematic nutritional intervention strategy that effectively prevents deficiency in pediatric population. Since 2008, the Pediatric Branch of the Chinese Medical Association has successively issued guidelines, initially recommending that infants take 400 IU of vitamin D supplementation daily starting 2 weeks after delivery (23), and raising the recommendation to 400–800 IU in 2021 (24), with additional supplementation recommended for children with limited sun exposure. National healthcare doctors provide vitamin D supplements for children based on this recommendation. Moreover, the increased awareness of parents regarding their children’s health is also an important factor. They are more informed about the function and importance of vitamin D supplementation, more willing to follow doctors’ recommendations for supplementation, and regularly monitor 25(OH)D concentrations to ensure adequate vitamin D status are achieved (12). In addition, the rapid development of China’s economy has significantly improved residents’ living standards. Foods high in vitamin D, such as marine fish, pig liver, and nutritionally enhanced foods like infant formula, milk, and orange juice have become more accessible, providing children with sufficient vitamin D (19, 25). These measures have significantly reduced the rate of vitamin D deficiency. Our research had shown that the vitamin D nutritional status of 1-year-old children is satisfactory, with an extremely low deficiency rate of only 0.5%, which is powerful evidence of the intake of fortified foods and supplements. Furthermore, thanks to the continuous advancements in vitamin D detection technology, the detection accuracy is constantly increasing. LC–MS/MS measured serum vitamin D forms accurately (26), validated by China’s external quality assessment program for reliable results. This finding indicates a potential shift in vitamin D sources from mainly self-synthesis to a balanced combination of artificial supplementation and natural synthesis.

We further analyzed the effects of COVID-19-related restrictions, and found minimal impact on vitamin D concentrations in children. The result was a surprise to researchers and reinforced that the concentrations of 25(OH)D were not linked to sunlight or COVID-19 infection reported in Italy (27). Although winter sunlight intensity significantly decreased and outdoor activities were reduced during COVID-19 lockdown management, which could impact vitamin D generation (28), standardized supplements and fortified food intake maintained relatively constant vitamin D status, effectively offsetting potential seasonal sunlight exposure fluctuations (29). An Italian research reported by Ferrari et al. (27) supports this speculation. In addition, to maintain children’s health, families have begun to make more use of outdoor spaces such as balconies and courtyards, as even brief sun exposure can help children synthesize vitamin D (29).

We showed that the concentrations of 25(OH)D gradually decrease with age, particularly in the 3–6 year old participants. In accordance with this, Yu et al. (18) revealed that 25(OH)D concentrations were lower in children aged 3 to 6 years compared to those under 3 years. And Isa et al. (30) also found that the concentrations of 25(OH)D were inversely related to the age of the participants. This decline is attributed to a combination of reasons, including diminished solar radiation, vitamin D supplements reduction, and reduced consumption of vitamin D from the diet. As children over 3 years enter the preschool stage, indoor activities increase, while sunlight exposure and outdoor exercise decrease, leading to a reduction in their own vitamin D synthesis (12). Although pediatric vitamin D guidelines recommend daily supplementation of at least 400 IU after birth (24), multiple factors contribute to gradually decreasing supplementation compliance with age (31), further exacerbating the vitamin D deficiency problem.

Additionally, in contrast to other seasons, winter had the lowest concentrations of 25(OH)D, a finding echoed by Wu Y (12). Similarly, Nakano et al. reported a marked decrease in vitamin D concentrations among Japanese children during winter (32). This decline in vitamin D concentrations in winter may result from reduced sunlight exposure (33), increased clothing coverage, and variations in latitude and geographical location (34). Likewise, the Hanzhong area (33.0° N, 107.0° E) is located in the Northern Hemisphere, experiencing insufficient sunlight and low UV light intensity in winter. Moreover, the seasonal variations in its concentrations are minimal. Considering the possibility that children in our research obtained vitamin D through supplements and sunlight, regular vitamin D supplementation might offset the influence of seasonal variations. In addition, we found that the serum concentrations of 25(OH)D_2_ were extremely low, about 1/19 of total concentrations, potentially reflecting the recommended types of vitamin D supplements for children. Although global consensus considers 25(OH)D_3_ and 25(OH)D_2_ equivalent in rickets prevention (35), some studies indicate that 25(OH)D_3_ is more potent (36). Consequently, vitamin D supplements for Chinese children typically contain only 500–700 units of vitamin D3, no vitamin D2 is included. In spring and summer, the concentrations of 25(OH)D_2_ were higher. Possibly due to the consuming sun-dried mushrooms, which are an important source of vitamin D_2_ (37). For picky or low-appetite children, sun-dried mushrooms are an option for obtaining vitamin D.

Our study reveals that the serum concentrations of 25(OH)D among children aged 1 to 6 years fluctuate with age and season, with a noticeable decline in children aged 3–6 years. It is recommended to increase outdoor activities in winter, ensure regular vitamin D supplementation, and check vitamin D concentrations when necessary. Although the study involved multiple participants, it was limited to a single center. Future research should validate our findings with a larger sample across different sites and consider a third sampling that incorporates additional covariates controlling for vitamin D, such as dietary habits, ethnicity, and lifestyle. These methods will enhance the generalizability of the results.

## 5 Conclusion

The research indicated that vitamin D deficiency is uncommon among Chinese children, with 25(OH)D concentrations experiencing a notable decline in those aged 3–6 years. The findings suggested a potential need for tailored supplementation strategies and possibly higher doses for this age group, along with monitoring 25(OH)D concentrations to evaluate supplementation effectiveness. COVID-19-related restrictions minimally affected children’s 25(OH)D concentrations, revealing the nutritional implications of the pandemic.

## Data Availability

The original contributions presented in the study are included in the article/Supplementary material, further inquiries can be directed to the corresponding author.
